# Editorial: Forest microbiome: dynamics and interactions in the Anthropocene era, volume II

**DOI:** 10.3389/fmicb.2026.1974582

**Published:** 2026-09-08

**Authors:** Amrita Chakraborty, Amit Roy, Shulin He, Antonio Castellano-Hinojosa, Fred O. Asiegbu

**Affiliations:** 1 Faculty of Forestry and Wood Sciences, Czech University of Life Sciences Prague, Prague, Czechia; 2 Chongqing Normal University, Chongqing, China; 3 Department of Microbiology, University of Granada, Granada, Spain; 4 Faculty of Agriculture and Forestry, University of Helsinki, Helsinki, Finland

**Keywords:** disturbances, environmental gradients, forest ecosystem, host-microbe interactions, soil microbiome, vegetation

## Introduction

Forest ecosystems are among the most biologically diverse terrestrial systems, relying on complex interactions between plants, soil, and microorganisms, and collectively regulating nutrient cycling, carbon sequestration, ecosystem productivity, and resilience to environmental change (Baldrian, 2017). Advances in high-throughput sequencing, ecological network analyses, and machine-learning approaches increasingly show that microbial communities are not merely indicators of ecosystem condition but active participants in the processes that determine forest stability and recovery. Yet fundamental questions remain about how forest composition, environmental gradients, disturbance regimes, and management interventions shape microbial diversity, community assembly, and function.

**Figure 1:**
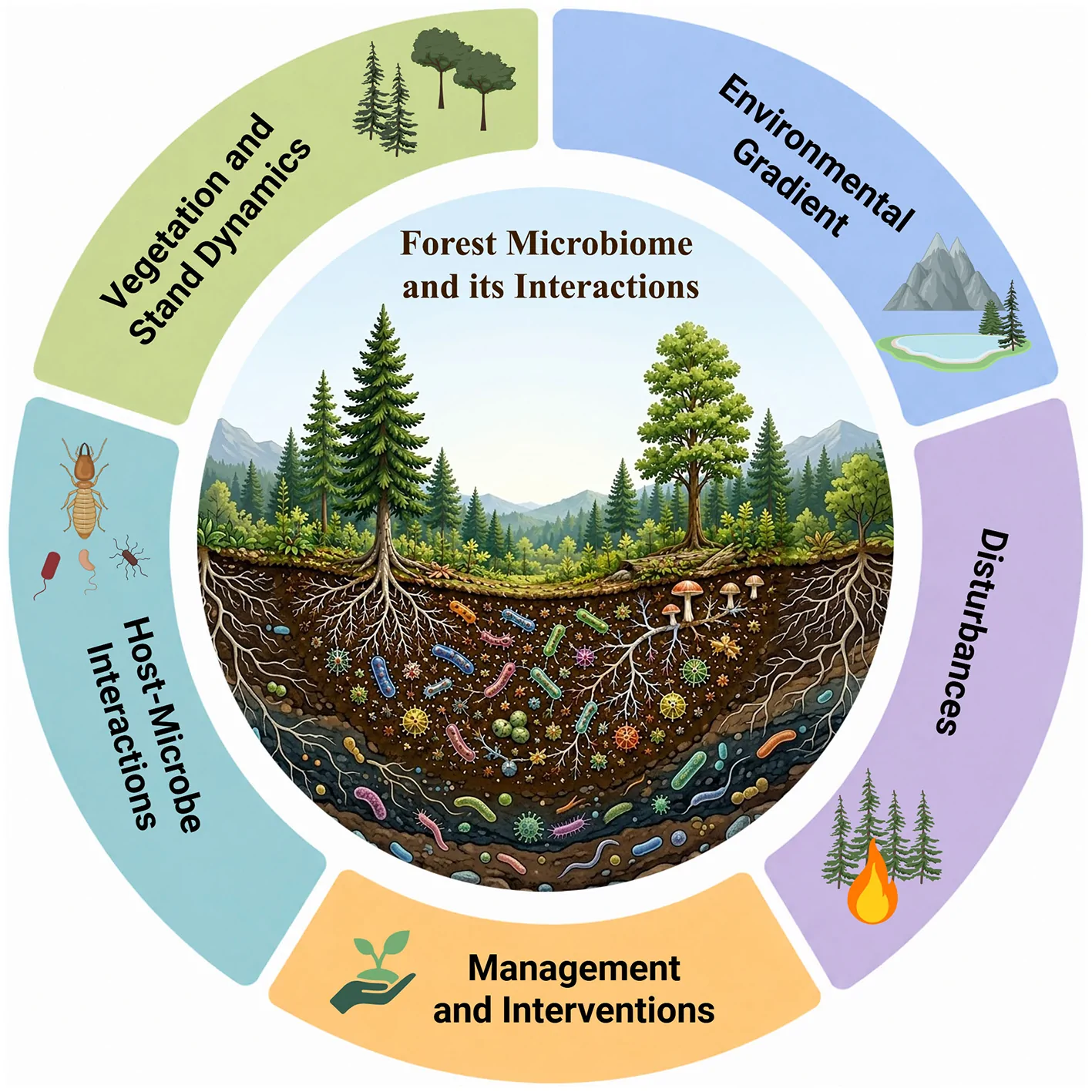
Conceptual framework of the major drivers shaping the forest microbiome. Forest microbiome is a dynamic network of microorganism and their interactions with plants, animals, soil, and the surrounding environment, collectively influencing ecosystem diversity and functioning. Microbial community composition and activity are shaped by different interconnected drivers such as vegetation and stand dynamics, including plant community composition, stand structure, succession, and root-associated processes; environmental gradients, including climate, topography, soil physicochemical properties, and resource availability; disturbances like forest fire and other natural or anthropogenic perturbations; management and interventions, which modify vegetation, soil conditions, and ecosystem processes; as well as host-microbe interactions. Together, these interacting factors regulates the diversity, composition, interactions, and functional potential of forest microbiomes, with consequences for nutrient cycling, plant performance, soil processes, ecosystem resilience, and forest functioning.

The studies featured in this Research Topic address these questions across diverse ecological settings, including coastal islands, karst landscapes, subtropical plantations, boreal forests, alpine ecosystems, Mediterranean woodlands, and insect hosts. Collectively, they demonstrate that microbial responses are shaped by the interplay between biological diversity and environmental context (Figure 1). Several recurring patterns emerge across the collection: vegetation composition and stand development drive the reorganization of microbial communities; soil nutrient availability and depth influence microbial functional potential; disturbance intensity can redirect community assembly and biogeochemical cycling; and targeted management can promote beneficial microbiomes and ecosystem multifunctionality. Together, these complementary perspectives advance our current understanding of the ecological influence and functional significance of forest microbiomes.

## Forest diversity and environmental gradients shape microbial communities

Vegetation diversity and forest composition emerged as major determinants of belowground microbial communities. Tree species modify litter inputs, root exudates, nutrient availability, and soil physicochemical conditions, thereby creating distinct microbial niches. Wu et al. demonstrated that mixed plantations of *Cryptomeria fortunei* and *Liquidambar formosana* in the karst regions of Guizhou Province, China, significantly reshaped bacterial and fungal community composition while increasing the abundance of beneficial bacterial groups involved in nutrient cycling. These findings support that mixing functionally contrasting tree species can broaden the diversity of carbon substrates and nutrient niches available in the soil, thereby influencing belowground microbial communities and ecosystem functioning. Similarly, Qiao et al. demonstrated that mixed oak-pine forests supported greater microbial diversity, stronger microbial interaction networks, and improved rhizosphere nutrient conditions compared with monoculture plantations. These findings indicate that vegetation diversification can increase belowground habitat heterogeneity and support microbial coexistence and functional complementarity.

Forest developmental stage further restructures microbial communities. He et al. examined *Pinus armandii* plantations along a stand-age chronosequence in a karst mountain ecosystem and found that stand age exerted a stronger influence on microbial community structure than the soil compartment. Bacterial and fungal communities exhibited contrasting assembly mechanisms, while rhizosphere microbial networks were more intricately and densely connected than those in bulk soil, highlighting the importance of considering microbial kingdoms and soil compartments separately.

Environmental gradients further modify these vegetation effects on microbial communities. Zhang et al. demonstrated that the soil microbiome associated with *Heliotropium arboreum* varied among coastal islands and soil depths, with pH and the availability of nitrogen and phosphorus influencing community composition. Likewise, Qiu et al. resolved soil fungi into habitat generalists, specialists, and opportunists and showed that broad-leaved plantations supported the highest diversity across habitat-specialization groups, supporting that vegetation-driven environmental heterogeneity can shape both fungal diversity and interaction structure during restoration.

Moving beyond community composition, Su et al. integrated metagenomics and machine learning to investigate microbial carbon fixation across subtropical forests differing in tree-species richness. The study revealed non-linear relationships among tree diversity, soil organic carbon, nitrate availability, and microbial carbon-fixation genes. Notably, moderate tree diversity produced the strongest stimulation of microbial carbon fixation, while responses differed substantially between topsoil and subsoil, underscoring the importance of soil depth when evaluating belowground ecosystem functions. These findings demonstrate that forest diversity does not enhance microbial functions in a simple, linear manner with increasing species richness but instead influences microbial processes through complex, nutrient-mediated mechanisms that vary with nutrient availability, soil depth, and specific metabolic pathways.

## Disturbance and management reshape belowground ecosystems

Disturbance represents another major driver of microbial community dynamics. Fire ranks among the most influential disturbances affecting forest soils, with impacts that scale strongly with severity. In *Larix gmelinii* forests, Jia et al. demonstrated that fire intensity determined post-fire microbial responses. Low-intensity fire temporarily increased bacterial diversity, whereas severe fire reduced bacterial diversity and richness; both intensities declined fungal diversity. Fire also altered soil carbon and nitrogen pools, as well as nitrogen-cycling functional genes, emphasizing that disturbance severity is critical for predicting microbial recovery and biogeochemical consequences.

Anthropogenic disturbance can similarly reorganize microbial assembly. Along a human-footprint gradient in alpine soils of the southeastern Tibetan Plateau, Liu et al. found that increasing human disturbance altered soil nutrient availability and microbial assembly while reducing ecosystem stability, despite greater microbial network complexity, a decoupling between topological complexity and functional robustness also reported in other disturbance-stressed soils. This apparent contrast features an important conceptual point: microbial network complexity alone should not be interpreted as evidence of ecological stability or ecosystem health.

Other contributions demonstrate that management can intentionally influence forest soil microbiomes. Ding et al. reported that alfalfa mulching in *Camellia oleifera* forests on a subtropical karst slope improved soil health and ecosystem multifunctionality, increased beneficial microbial groups, and reduced potentially pathogenic fungi. González-Reguero et al. extended this management perspective by combining plant growth-promoting bacteria with organic fertilizers derived from wastewater treatment residues to improve cork oak establishment under controlled conditions. The treatment improved seedling performance while maintaining rhizosphere microbial structure and function, linking microbial biotechnology with reforestation and circular-bioeconomy approaches. However, field validation will be important for determining the broader applicability of these responses.

Although most contributions in our current Research Topic focus on soil microbiomes, Purohit et al. broaden the ecological perspective by investigating host-associated bacterial communities in two economically important subterranean termite species. The two species harbored distinct bacterial assemblages but shared a core microbiota dominated by Spirochaetota. Eugenol exposure caused termite mortality and selectively reshaped bacterial community composition rather than causing wholesale microbiome collapse. This work illustrates the ecological specificity of host-microbe associations and suggests that selective perturbation of symbiotic microbiota may inform lower-impact pest-management approaches, while also underscoring the need to understand functional consequences of microbiome disruption before such strategies are translated into practice.

## Conclusion and future perspectives

Despite the ecological breadth, several common conclusions emerge from the collection of articles in this Research Topic. First, taxonomic diversity alone cannot adequately explain ecosystem functioning. Functional genes, interaction networks, community assembly mechanisms, soil physicochemical properties, and ecosystem multifunctionality provide complementary information about belowground processes. Second, bacterial and fungal communities often respond differently to the same environmental driver, making multi-kingdom analyses essential for understanding forest microbiome dynamics (Bahram et al., 2018). Third, microbial responses are strongly context dependent: forest composition, stand age, soil compartment and depth, nutrient availability, disturbance intensity, and management regime can all change the direction or magnitude of observed effects. Finally, restoration and management can deliberately influence microbiomes, but successful application requires matching interventions to local ecological conditions rather than assuming universally beneficial microbial responses.

The studies presented here, therefore, position forest microbiomes as integral components of ecosystem resilience rather than passive indicators of soil quality. At the same time, they expose a persistent gap between describing microbial community structure and establishing causal links to ecosystem processes; closing this gap remains the fundamental challenge for the field. Future work should combine state-of-the-art omics technologies, such as metagenomics, metatranscriptomics, and metabolomics, with targeted functional assays and long-term, manipulative field experiments to establish the relationship between microbial communities and ecosystem processes. Greater attention is also needed to temporal dynamics, vertical soil stratification, interactions among microbial kingdoms, and connections among rhizosphere, bulk soil, litter, and host-associated microbiomes. Such approaches, along with modeling, will help distinguish microbial signatures that merely correlate with ecosystem condition from microbial mechanisms that can reliably predict or improve forest ecosystem function.

As forests face accelerating climate change, increasingly complex disturbance regimes, and intensifying land-use pressures, effective restoration and sustainable management will require moving beyond vegetation-centered approaches to incorporate soil microbiomes into ecological planning and conservation frameworks explicitly (Asiegbu et al., 2026). The contributions in this Research Topic demonstrate that microbial communities are integral to nutrient cycling, carbon dynamics, soil health, plant performance, and ecosystem stability, while responding dynamically to environmental change, disturbance, and management practices (Asiegbu and Kovalchuk, 2021). Integrating these belowground processes into monitoring, restoration planning, and adaptive forest management can provide a stronger mechanistic basis for enhancing forest resilience and sustaining ecosystem functioning under a changing environment.
